# Investigation of the Pharmacological Effect and Mechanism of Jinbei Oral Liquid in the Treatment of Idiopathic Pulmonary Fibrosis Using Network Pharmacology and Experimental Validation

**DOI:** 10.3389/fphar.2022.919388

**Published:** 2022-06-15

**Authors:** Aijun Zhang, Yixuan Zou, Qingcui Xu, Shuo Tian, Jie Wang, Yilin Li, Renchao Dong, Liangzong Zhang, Juanjuan Jiang, Lili Wang, Kai Tao, Zhaoqing Meng, Yanqiu Liu

**Affiliations:** ^1^ Institute of Chinese Materia Medica, Shandong Hongji-tang Pharmaceutical Group Co., Ltd., Jinan, China; ^2^ Shandong University of Traditional Chinese Medicine, Jinan, China

**Keywords:** idiopathic pulmonary fibrosis, jinbei oral liquid, network pharmacology, MAPKS, pulmonary function, E-cadherin, vimentin

## Abstract

**Overview:** Idiopathic pulmonary fibrosis (IPF) is a disease caused by many factors, eventually resulting in lung function failure. Jinbei oral liquid (JBOL) is a traditional Chinese clinical medicine used to treat pulmonary diseases. However, the pharmacological effects and mechanism of the action of JBOL on IPF remain unclear. This study investigated the protective effects and mechanism of the action of JBOL on IPF using network pharmacology analysis, followed by *in vivo* and *in vitro* experimental validation.

**Methods:** The components of JBOL and their targets were screened using the TCMSP database. IPF-associated genes were obtained using DisGeNET and Drugbank. The common targets of JBOL and IPF were identified with the STRING database, and a protein–protein interaction (PPI) network was constructed. GO and KEGG analyses were performed. Sprague–Dawley rats were injected with bleomycin (BLM) to establish an IPF model and treated orally with JBOL at doses of 5.4, 10.8, and 21.6 ml/kg. A dose of 54 mg/kg of pirfenidone was used as a control. All rats were treated for 28 successive days. Dynamic pulmonary compliance (Cdyn), minute ventilation volume (MVV), vital capacity (VC), and lung resistance (LR) were used to evaluate the efficacy of JBOL. TGF-β–treated A549 cells were exposed to JBOL, and epithelial-to-mesenchymal transition (EMT) changes were assessed. Western blots were performed.

**Results:** Two hundred seventy-eight compounds and 374 targets were screened, and 103 targets related to IPF were identified. Core targets, including MAPK1 (ERK2), MAPK14 (p38), JUN, IL-6, AKT, and others, were identified by constructing a PPI network. Several pathways were involved, including the MAPK pathway. Experimentally, JBOL increased the levels of the pulmonary function indices (Cdyn, MVV, and VC) in a dose-dependent manner and reduced the RL level in the BLM-treated rats. JBOL increased the epithelial marker E-cadherin and suppressed the mesenchymal marker vimentin expression in the TGF-β–treated A549 cells. The suppression of ERK1/2, JNK, and p38 phosphorylation by JBOL was validated.

**Conclusion:** JBOL had therapeutic effects against IPF by regulating pulmonary function and EMT through a systemic network mechanism, thus supporting the need for future clinical trials of JBOL.

## Introduction

Idiopathic pulmonary fibrosis (IPF) is a severe lung disease with the pathological characteristics of greatly diminished lung function, excessive fibroblast proliferation, and extracellular matrix deposition (Meyer 2017; Kim, Kugler et al., 2018). The 5-year survival rate for IPF is less than 30%. The pathogenetic mechanisms underlying IPF have not been fully established. Thus, the current drugs used to treat mild-to-moderate IPF, such as pirfenidone (PFD) and nintedanib, have limited benefit due to their failure to prolong the survival of IPF patients (Mazzei ME 2015). Moreover, PFD produces several adverse side effects, including gastrointestinal reactions, rashes, and photosensitivity (Antoniu, 2006). Therefore, novel drugs to effectively treat IPF need to be developed.

Jinbei oral liquid (JBOL) is a traditional Chinese herbal medicine (Tao K 1997, Tao, Zheng et al., 2013). It is composed of 12 herbs, *Astragalus membranaceus* (Fisch.) Bge, *Codonopsis pilosula* (Franch.) Nannf, *Glehnia littoralis* Fr. Schmidt ex Miq, *Salvia miltiorrhiza* Bge, *Angelica sinensis* (Oliv.) Diels, *Ligusticum striatum* DC, *Fritillaria cirrhosa* D. Don, *Pinellia ternata* (Thunb.) Breit, *Glycyrrhiza uralensis* Fisch, *Lonicera japonica* Thunb, *Scutellaria baicalensis* Georgi, and *Forsythia suspensa* (Thunb.) Vahl. (Zhang, Jie et al., 2012; Yu, Yang et al., 2020). JBOL is used to treat pulmonary interstitial fibrosis and acute lung injury (Zhang, Jie et al., 2012; Zhang, Wang et al., 2013). Most of the 12 herbs have been reported to have anti-inflammatory, antioxidative, antiviral, and immunomodulatory activities (Ng, Liu et al., 2004; Qi, Gao et al., 2017; Li Y 2021). Our previous study demonstrated that JBOL ameliorated bleomycin (BLM)-induced IPF in rats by inhibiting the production of inflammatory factors (Zhang, Cui et al., 2018; Xing, Qiang et al., 2020). However, the mechanism of action of JBOL on IPF is still largely unknown.

Network pharmacology is a valuable strategy to elucidate the mechanisms of action of traditional Chinese medicines (TCM), especially to specify their characteristics, including multiple components and numerous pathway targets (Hopkins and Andrew 2007; Shao and Bo 2013). The approach taken in this study utilized network analysis, combinations of targets, bioinformatics, and analysis of structure–activity relationships and connectivity. The results offered a new paradigm for verifying TCM efficacy and systematic determination of the function of different chemical components (Liu, Wang et al., 2013). Although network pharmacology was first described for drug discovery, its successful application in TCM studies has been explored by Professor Shao Li and others (Shao and Bo 2013). The network pharmacology of several Chinese medicines has been elucidated, including the mijianchangpu decoction (Xz, Jla et al., 2021), Moluodan (Zhou, Zhang et al., 2022), and Sinisan (Wei, Hou et al., 2021).

In the present study, an integrated investigation of JBOL was employed using active ingredient screening, target prediction, and network construction. The pharmacological mechanisms of JBOL on IPF and its potential targets were verified using ***in vitro*** and ***in vivo*** experimental methods.

## Materials and Methods

### Network Pharmacology

#### Screening JBOL Chemical Components

The Traditional Chinese Medicine System Pharmacology (TCMSP) Database, http://lsp.nwu.edu.cn/tcmsp.php) was used. Additional reports were identified using the PubMed database searching the plant names in references that were published in the last 10 years. The JBOL active compounds were screened based on the properties of absorption, distribution, metabolism, and excretion (ADME). Two ADME-related models were developed. 1) Oral bioavailability (OB) is a critical pharmacokinetic parameter representing the percentage of a dose of an orally administered drug that reaches the systemic circulation. 2) DL is an index used to estimate the “drugability” (the ability of a compound to be used as a pharmaceutical drug) of potential drugs and is calculated using the Tanimoto coefficient. An OB > 20% and DL > 0.10 were used as criteria in the TCMSP screening. We also screened the available literature using the Latin names of the 12 herbs as keywords in the PubMed database to identify additional chemical components.

### Screening Possible JBOL Targets

The drug–target network was mapped based on the source of the TCMSP database. For experimental validation of the compound targets, information was retrieved from the HIT database (Ye, Ye et al., 2011). Compounds without validated targets were screened using SysDT, a previously developed model that efficiently combines chemical and pharmacological information for drug targets using the random forest (RF) and support vector machine (SVM) algorithms. This method has demonstrated incredible success in predicting drug–target interactions. A previous report indicated the specificity was 93%, concordance was 82%, and sensitivity was 81% (Yu, Chen et al., 2012). Based on the aforementioned methods, target information for JBOL compounds was obtained from the TCMSP database.

### Screening for Possible IPF Targets

Target genes related to IPF were collected by searching the keyword “IPF” in the DisGeNET (https://www.disgenet.org/) (Paolacci, Precone et al., 2019) and DrugBank (Wishart, Feunang et al., 2017) (https://www.drugbank.ca/) databases. Genes identified in DisGeNET were scored, and the IPF-related genes were screened based on scores greater than the median score (Zhang, Li et al., 2019).

### Overlapping JBOL and IPF Targets

Bioinformatics (http://www.bioinformatics.com.cn/) (Zhou, Zhou et al., 2019) was used to screen the overlapping targets between the JBOL compound targets and the IPF-related targets. The overlapping JBOL and IPF targets were utilized to construct the network and conduct additional analyses.

### Protein–Protein Interaction Network Construction for the Overlapping Targets

The STRING database (https://string-db.org/) was the primary tool used to construct the PPI network. The interaction relationships between the functional proteins were included in this database (Franceschini, Szklarczyk et al., 2013; Lv, Xu et al., 2020). *Homo sapiens* was selected as the species of interest. The overlapping target proteins were imported, and the interaction information was analyzed. PPIs were selected based on a confidence score greater than 0.95 then the network was constructed and analyzed.

### Gene Ontology and Kyoto Encyclopedia of Genes and Genomes Analyses

GO and KEGG analyses were performed based on the Metascape System (https://metascape.org/). The GO analysis of the biological processes, molecular functions, cell component annotation, and the KEGG pathway enrichment analysis were performed based on a *p-*value < 0.05 (Ashburner, Ball et al., 2000).

### Network Construction

Cytoscape 3.2.1 software was employed to construct and visualize the networks, including the 12 herb–compound–target networks, the PPI network for overlapping targets for JBOL compounds and IPF targets, and the pathway–target network. All networks were plotted, and the network nodes indicated herbs, compounds, or target proteins. The edges represented interactions between compounds, targets, and pathways. The color and node size were proportional to the degree value, which was defined based on the number of connecting edges.

### Experimental Verification

**FIGURE 1:**
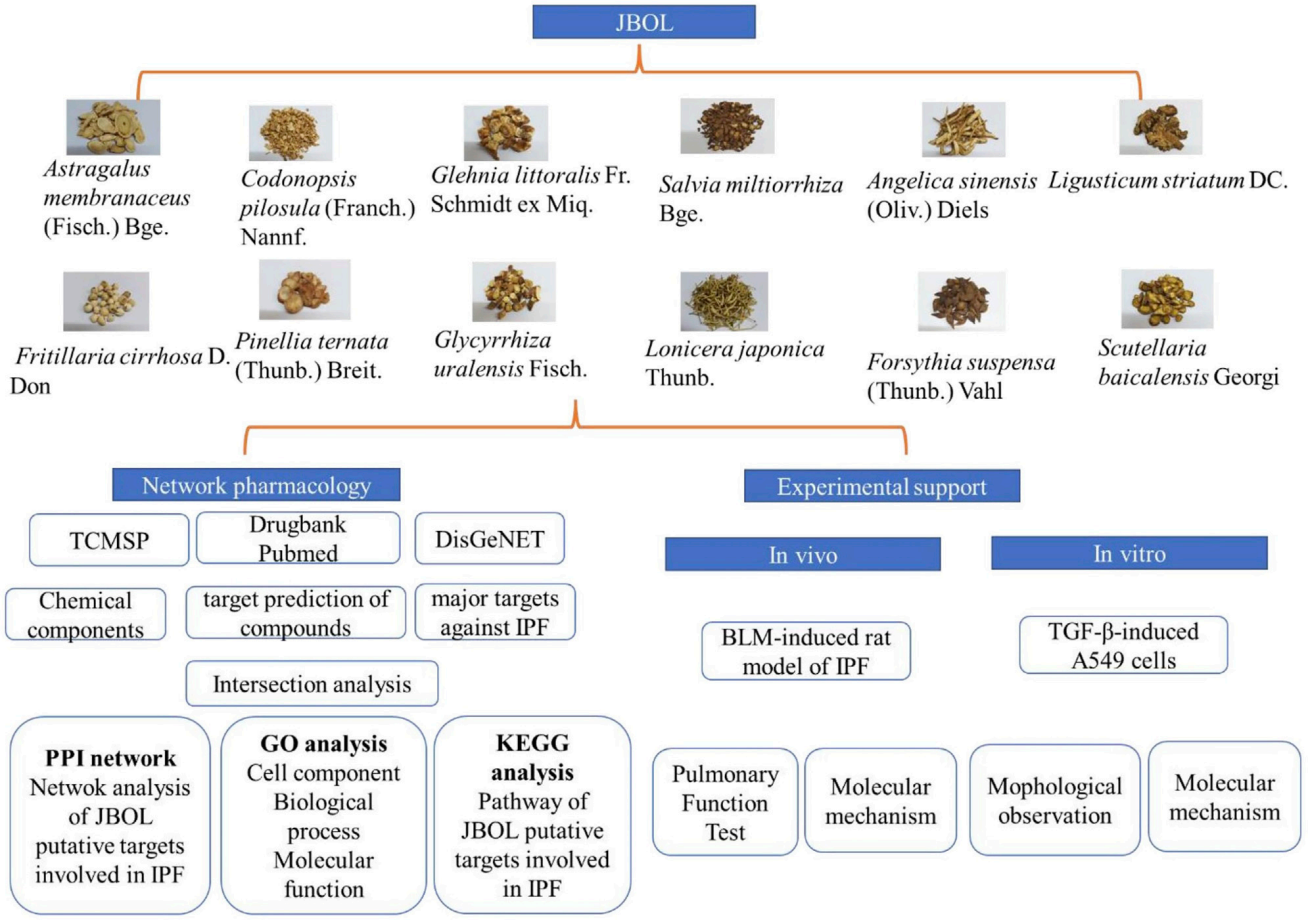
The research workflow. Compounds from JBOL were identified based on the TCMSP database considering their drug-likeliness and oral bioavailability. The targets also were collected. Then, the IPF-related targets were screened using the DisGeNET database. The overlapping JBOL targets and IPF targets were used to construct the PPI network with the STRING database. Metascape was used to analyze the GO results and the KEGG pathway enrichment. The herb–compound–target network was constructed using Cytoscape. Experimental validation of the rat and cell culture models was performed to confirm the mechanism of action of JBOL.

The study workflow is illustrated in Figure 1.

## Materials and Reagents

Jinbei oral liquid (JBOL) (batch number: 2102001) was provided by Shandong Hongjitang Pharmaceutical Co., Ltd. (Jinan, China). The doses of each herb in 1,000 ml of JBOL were as follows: 66 g *Astragalus membranaceus*, 66 g *Codonopsis pilosula*, 70 g *Glehnia littoralis*, 66 g *Salvia miltiorrhiza*, 55 g *Angelica sinensis*, 55 g *Ligusticum striatum*, 46 g *Fritillaria cirrhosa*, 46 g *Pinellia ternata*, 35 g *Glycyrrhiza uralensis*, 115 g *Lonicera japonica*, 46 g *Scutellaria baicalensis*, and 46 g *Forsythia suspensa*. The plant names were checked on http://www.theplantlist.org. Bleomycin (BLM) was purchased from the Hanhui Pharmaceuticals Company (Shanghai, China). Pirfenidone was obtained from the Beijing Continent Pharmaceuticals Company (Beijing, China). TGF-β1 was purchased from MedChemExpress (Shanghai, China). Antibodies, including E-cadherin, vimentin, phospho-P38, phospho-ERK1/2, phospho-JNK, and GAPDH, were obtained from Cell Signaling Technology (MA. USA). The reference compounds, including adenosine (110879-201703), guanosine (111977-201501), chlorogenic acid (110753-202018), loganin (111640-201808), ferulic acid (110773-201614), imperialine (110767-201710), peimine (110750-201311), peiminine (110751-201712), imperatorin (110826-201918), and 18β-glycyrrhetinic acid (110723-201715) were provided by the National Institutes for Food and Drug Control. Rutin (120025-202004), liquiritigenin (070002-202011), and formononetin (130035-202003) were purchased from Shandong WoDeSen Bioscience Technology, Ltd. (Jinan, China). Isoliquiritigenin (B21525) was obtained from Yuanye Biotech Co., Ltd. (Shanghai, China). Tanshinone I (17122704) was obtained from Chengdu Pufei De Biotech Co., Ltd. (Chengdu, China). The purity of all reference compounds was greater than 98%.

### UHPLC Analysis of JBOL

An Agilent 1290 UHPLC system (Agilent Technologies, Palo Alto, CA, United States) was used to analyze JBOL, and separation was achieved with a Waters ACQUITY UPLC^®^ BEH C18 column (2.1 **×** 100 mm, 1.7 μm) (Shanghai, China). The column temperature was 30°C. The mobile phase was an elution composed of 0.1% formic acid water (A) and acetonitrile (B). One-μl samples were injected into the system, and the mobile phase flow rate was 0.3 ml/min.

### Pulmonary Fibrosis Induced in Rats Using BLM

Sixty adult male Sprague–Dawley (SD) rats, weighing approximately 200 g, were obtained from the Vital River Laboratory Animal Technology Co., Ltd. (Beijing, China). The animal experiments were approved by the Animal Ethics Committee at Shandong University of Traditional Chinese Medicine. All rats were acclimated in an animal holding room at 25°C and provided food and water *ad libitum*. The rats were divided into six groups: 1) treatment with deionized water (control group); 2) treatment with 3 mg/kg BLM administered intratracheally (i.t, BLM group); 3) treatment with 3 mg/kg BLM (i.t.) and intragastric administration (i.g.,) of JBOL (5.4 ml/kg); 4) treatment with 3 mg/kg BLM (i.t.) and JBOL (10.8 ml/kg, i.g.); 5) treatment with 3 mg/kg BLM (i.t.) and JBOL (21.6 ml/kg, i.g.); 6) treatment with 3 mg/kg BLM (i.t.) and pirfenidone (54 mg/kg, i.g.) as a positive control. The doses of JBOL were chosen based on our previous reports (Zhang, Cui et al., 2018; Xing, Qiang et al., 2020). The compounds were administered once every day according to the aforementioned treatment groups. After 28 days of treatment, the rats were anesthetized, and the pulmonary function was assessed. Subsequently, the lung tissues were collected and frozen for protein expression assessment.

### Pulmonary Function Assay

The rats were anesthetized and fixed in a supine position on a board. A trachea tube was inserted into the trachea and connected with the body plethysmograph. The change in gas pressure was measured to calculate the lung volume indirectly. The indices, including dynamic pulmonary compliance (Cdyn), minute ventilation volume (MVV), vital capacity (VC), and lung resistance (LR) data were determined.

### Cell Culture and JBOL Treatment

Human type II alveolar epithelial A549 cells were purchased from the Type Culture Collection of the Chinese Academy of Sciences (Shanghai, China). Dulbecco’s modified Eagle’s medium (DMEM, Gibco, Paisley, United Kingdom) with 10% fetal calf serum (FCS), 100 μg/ml streptomycin, and 100 U/ml penicillin was used for cell culture. The A549 cells were maintained at 37°C with 5% CO_2_ in a humidified chamber.

The A549 cells were treated with culture medium (control) or 5 ng/ml TGF-β1 with or without 0.05, 0.5, or 1% JBOL. JBOL was diluted using a culture medium. After incubation for 48 h, the cells were observed using an inverted phase-contrast microscope (Olympus, Japan). The cell morphology, including characteristics of mesenchymal–epithelial transition (EMT), was observed, and images were captured at ×200 magnification. For the protein expression assay, JBOL and 5 ng/ml TGF-β1 were added to the cells and incubated for 72 h. Then the cells were lysed for analysis.

### Western Blot Assay

Lung tissue samples or A549 cells were treated for 40 min with RIPA lysis buffer containing phosphatase inhibitors, then centrifuged (12,000 rpm, 15 min) at 4°C. The protein concentration of the supernatant was assessed using bicinchoninic acid. Individual protein samples (10–30 μg) were loaded onto SDS-polyacrylamide gels for separation and then transferred to PVDF membranes (Millipore, Darmstadt, Germany). The membranes were incubated with different primary antibody solutions and shaken overnight. After the membranes were washed with Tris-buffered saline with Tween-20 (TBST), HRP-labeled secondary antibodies were added, and the membranes were incubated for 2 h. The protein bands were visualized using ECL-Plus detection (Biyuntian, Shanghai, China). The intensity of the protein bands was quantified using Image Pro-Plus 6.0 software.

### Statistical Analysis

All data were expressed as means ± standard error of the mean (SEM). The one-way analysis of variance (ANOVA) was used for multiple comparisons. The Student’s *t*-test was used to compare the differences between the two groups. A *p*-value < 0.05 was considered statistically significant.

## Results

### JBOL Compound Screening and Target Prediction

**FIGURE 2:**
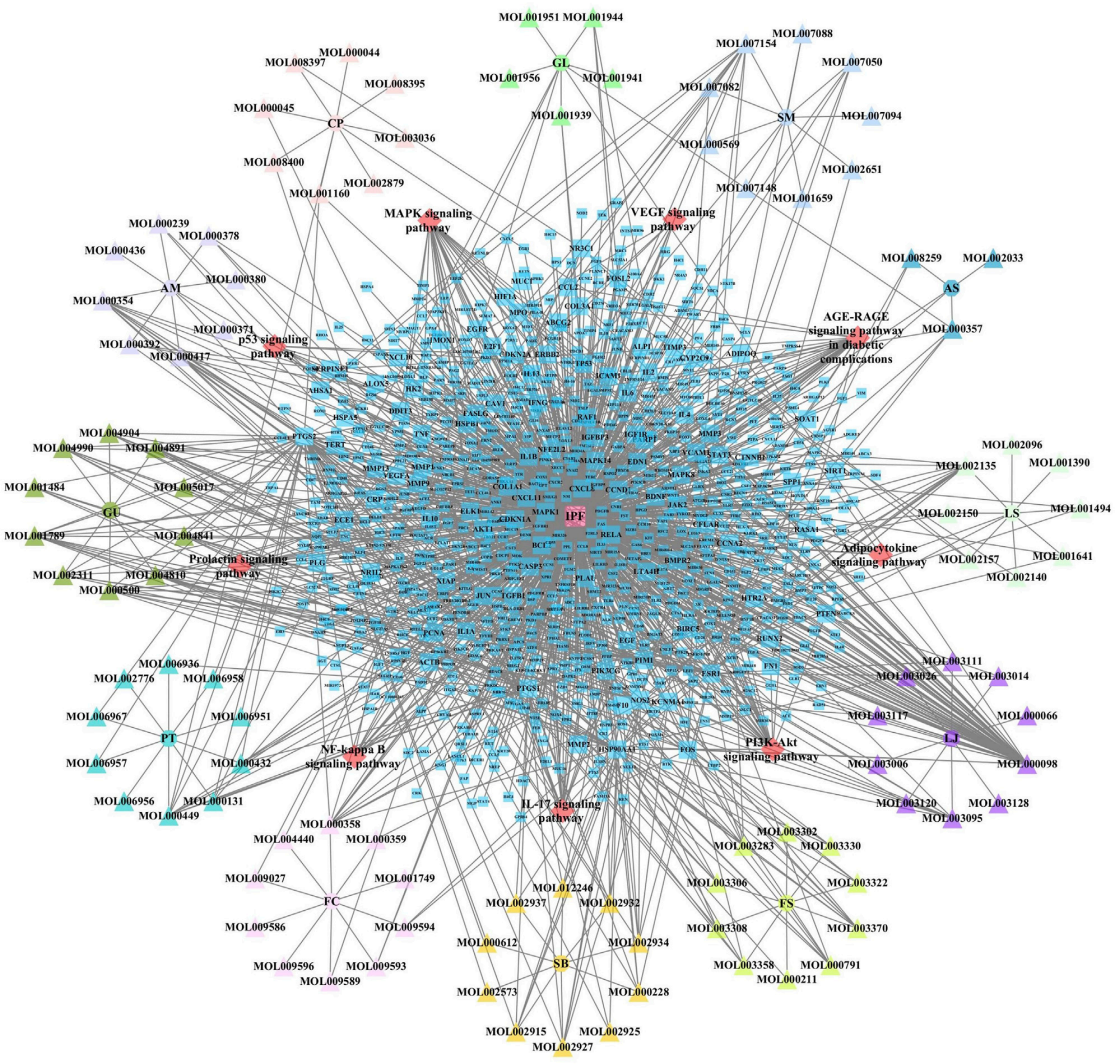
The compound–target network of JBOL. Circles represent herbs, triangles represent compounds derived from herbs, and squares represent the predicted targets. The node size indicates the value of the degree.

JBOL contained 12 herbs, including Huangqi (*Astragalus membranaceus,* AM), Dangshen (*Codonopsis pilosula,* CP), Beishashen (*Glehnia littoralis*, GL), Danshen (*Salvia miltiorrhiza,* SM), Danggui (*Angelica sinensis*, AS), Chuanxiong (*Ligusticum striatum,* LS), Chuanbeimu (*Fritillaria cirrhosa*, FC), Banxia (*Pinellia ternata*, PT), Gancao (*Glycyrrhiza uralensis*, GU), Jinyinhua (*Lonicera japonica*, LJ), Lianqiao (*Forsythia suspensa*, FS), and Huangqin (*Scutellaria baicalensis*, SB). The TCMSP database was used to determine the compounds present in JBOL and predict their targets. Two hundred seventy-eight candidate compounds were identified in JBOL, including 15 in AM, 20 in CP, 10 in GL, 27 in SM, 5 in AS, 9 in LS, 40 in FC, 29 in PT, 48 in RU, 15 in LJ, 22 in FS, and 97 in SB (Supplementary Table S1). The 278 compounds targeted 374 proteins (Supplementary Table S2). The screened compounds and their targets were mapped to generate the compound–target interaction network (Figure 2).

### Potential Targets for IPF

**FIGURE 3:**
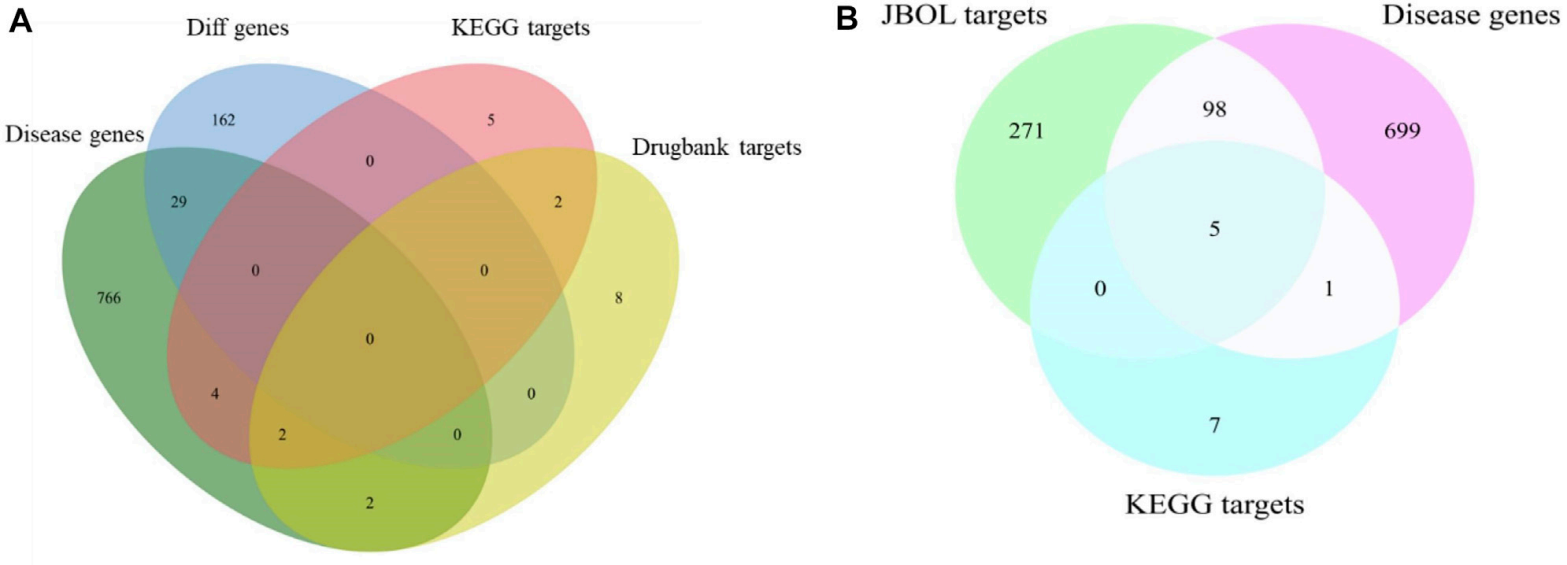
Overlap of the target genes between IPF and JBOL. **(A)** Matching for the IPF genes, KEGG, and drug bank genes. **(B)** Matching for the IPF genes, KEGG drug target genes, and JBOL target genes.

The IPF-associated genes and targets were obtained from the DisGeNET (https://www.disgenet.org/) and DrugBank databases (Supplementary Table S3). The genes that overlapped with the JBOL compound targets were identified (Figure 3). One hundred and three overlapping targets were found based on the degree of correlation between the JBOL targets and the IPF targets. Information concerning the overlapping targets is shown in Supplementary Table S4.

### Identifying Important JBOL Targets Using Intersection Analysis

**FIGURE 4:**
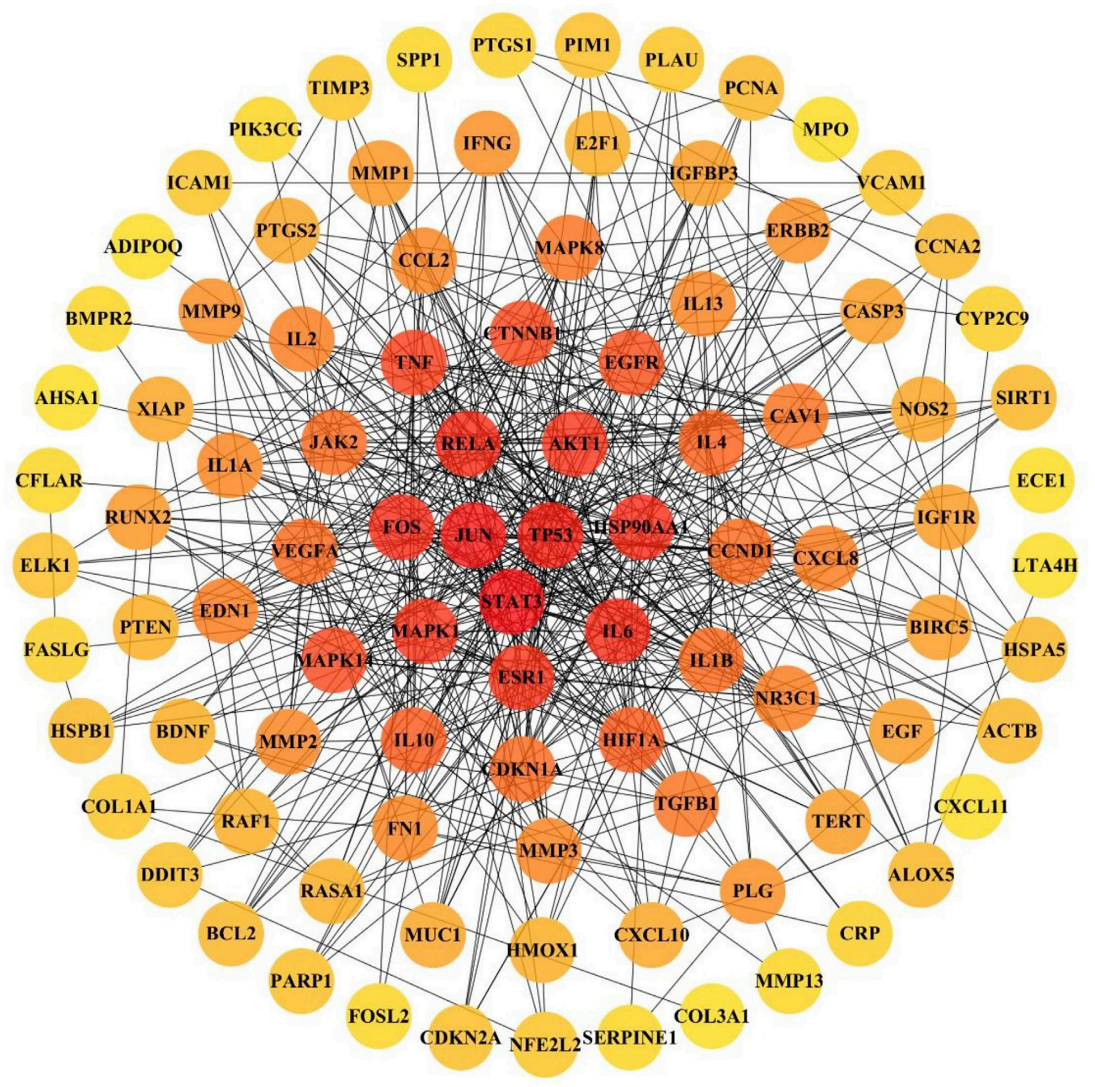
The PPI network of overlapping targets between IPF and JBOL. 103 overlapping targets were plotted as nodes. The edges represent the interaction relationships between the nodes. The darker node color indicates a higher degree value.

The 103 overlapping JBOL targets, IPF genes, and anti-IPF drug targets were analyzed based on the STRING database. The protein–protein interactions (PPI) network was constructed using a confidence score of 0.4. The PPI network nodes represented the selected targets, and the edges represented target interactions. As seen in Figure 4, the PPI network consisted of 103 nodes and 501 edges. The degree value represented the connection intensity. The average node degree was 9.63. The central nodes with many more edges possibly played essential roles in the JBOL treatment of IPF, including MAPK1 (ERK2), MAPK14 (p38), MAPK8 (JNK1), JUN, IL-6, and AKT.

### The GO and KEGG Enrichment Analyses

**FIGURE 5:**
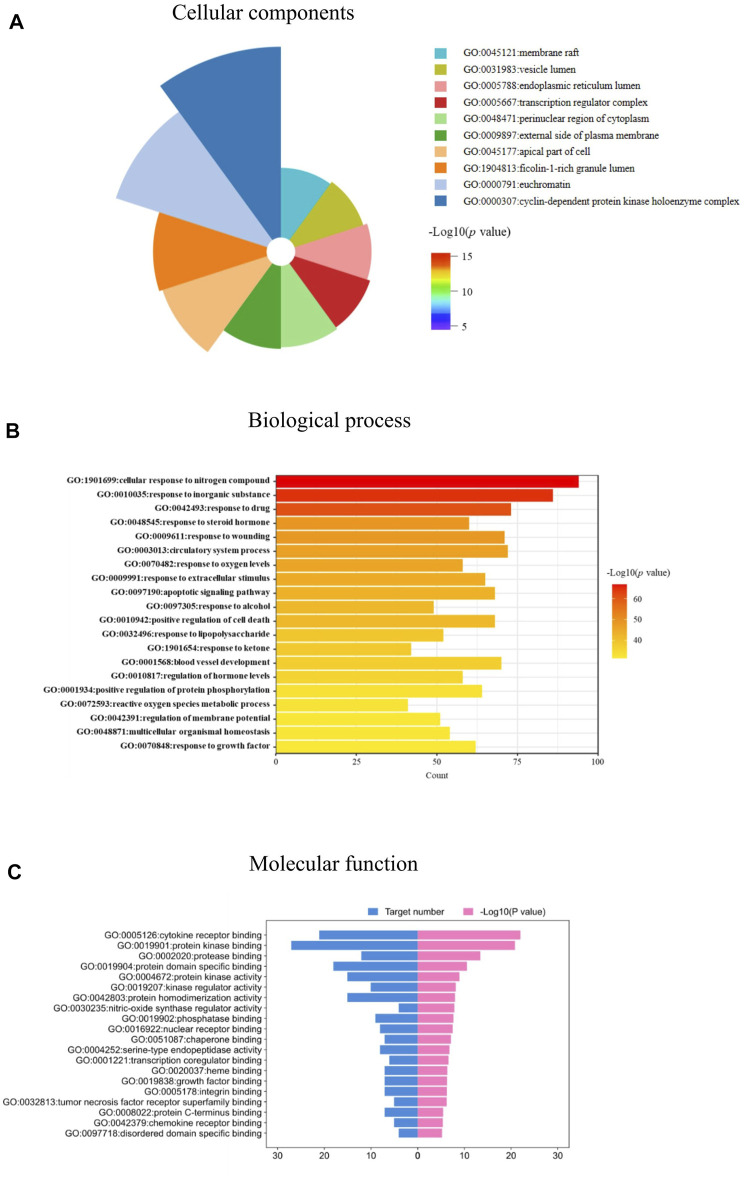
biological function analysis of the overlapping targets. **(A)** Cellular components analyses. **(B)** Biological processes analyses. **(C)** Molecular function analyses related to JBOL treatment of IPF.

The 103 targets were imported into the Metascape system for the GO and KEGG pathway analyses. The GO analysis revealed targets associated with numerous cellular components, biological processes, and molecular functions critical for drug development and IPF treatment. One hundred and eleven enriched cell components were identified, and the top 10 cell components were closely associated with vesicle lumen, membrane rafts, endoplasmic reticulum lumen, cyclin-dependent protein kinase holoenzyme complex, and transcription regulator complex (Figure 5A). In addition, based on a *p*-value < 0.05, 1,956 enriched biological processes were identified, including response to lipopolysaccharide, response to steroid hormones, regulation of oxygen levels, response to growth factors, and others (Figure 5B). The molecular function targets included cytokine receptor binding, protein kinase binding, kinase regulator activity, and growth factor binding (Figure 5C). The identified cellular components, biological processes, and molecular functions indicated the critical actions of JBOL in IPF treatment.

**FIGURE 6:**
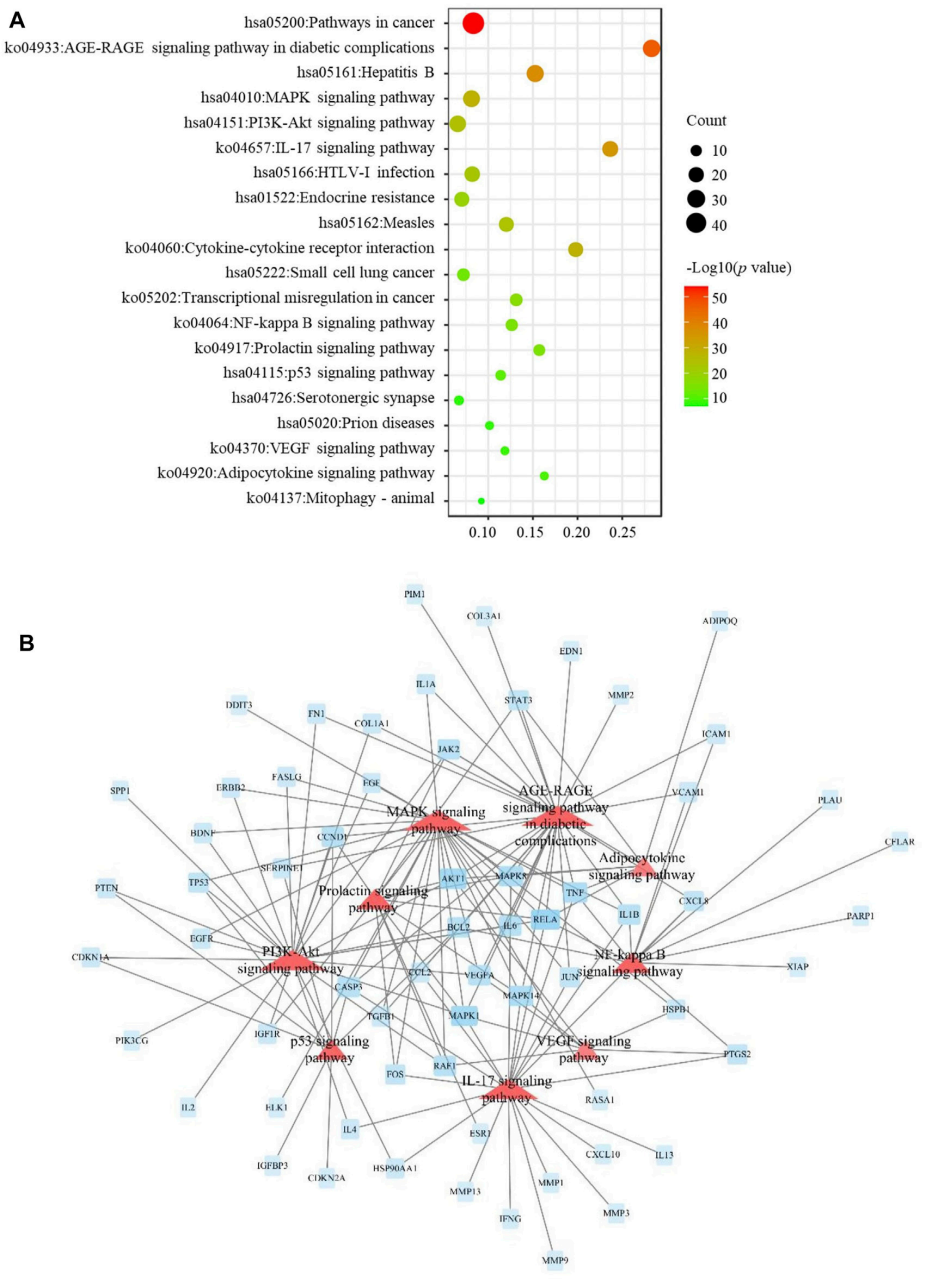
KEGG pathway enrichment analysis. **(A)** Signaling pathways related to the JBOL treatment of IPF. **(B)** The key pathways related to IPF regulated by JBOL.

To assess the molecular mechanism of JBOL in treating IPF in more detail, we enriched the KEGG pathways for the 103 targets. Forty-one pathways were identified based on a *p*-value < 0.05 (Figure 6A). These pathways included the MAPK signaling, cGMP-PKG, estrogen, thyroid hormone, HIF-1, cAMP, PI3K-Akt, IL-17, and AGE-RAGE pathways as well as others. A network involving these pathways was established to reveal their interaction relationships (Figure 6B).

### JBOL Protected Against Pulmonary Fibrosis in the Rat and A549 Cell Models

**FIGURE 7:**
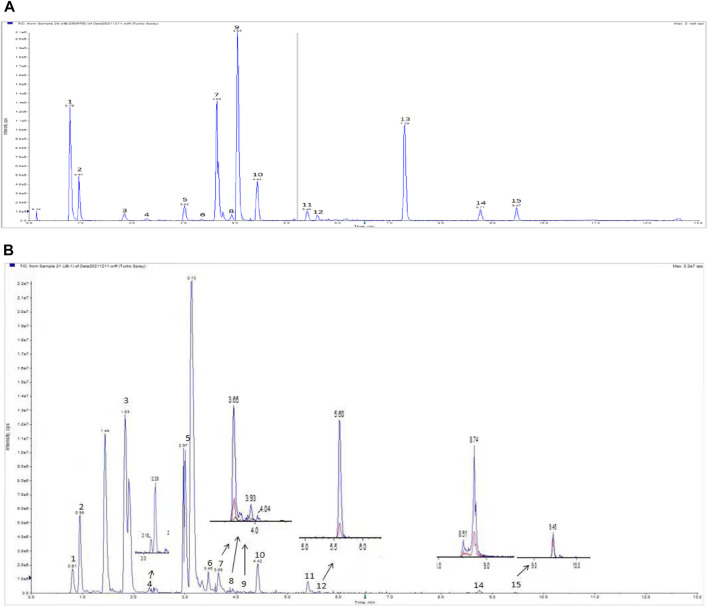
UHPLC analysis of JBOL. A chromatogram of mixed reference standards **(A)** and Jinbei oral liquid **(B)**. UPLC conditions: column 2.1 × 100 mm, 1.7 μm particle size. The mobile phase was composed of component A (0.1% formic acid in water) and component B (acetonitrile). The flow rate was 0.3 ml/min.

The BLM-induced rat model and the TGF-β1–induced A549 cell model were used to validate the efficacy and mechanism of JBOL actions on IPF. First, to monitor JBOL chemical stability, the major chemical components in JBOL were determined using the UHPLC analysis (Figure 7). Among the identified components, in addition to chlorogenic acid, which was present at high levels (Table 1), an additional seven components were identified in the TCMSP database, including guanosine, formononetin, liquiritigenin, isoliquiritigenin, imperatorin, tanshinone I, and 18β-glycyrrhetinic acid, based on the criteria of OB > 20% and DL > 0.10. Thus, these components were used as identifying components for JBOL (Supplementary Table S5).

**TABLE 1:** Determination of 15 detected compounds in JBOL.

No.	Compound	Content (μg/ml)
1	Adenosine	12.5
2	Guanosine	8.03
3	Chlorogenic acid	70.25
4	Loganin	3.87
5	Rutin	4.25
6	Ferulic Acid	7.00
7	Imperialine	0.30
8	Peimine	0.01
9	Peiminine	0.03
10	Liquiritigenin	3.25
11	Isoliquiritigenin	0.87
12	Formononetin	1.05
13	Imperatorin	0
14	Tanshinone I	0.28
15	18β-Glycyrrhetinic Acid	0.23

**FIGURE 8:**
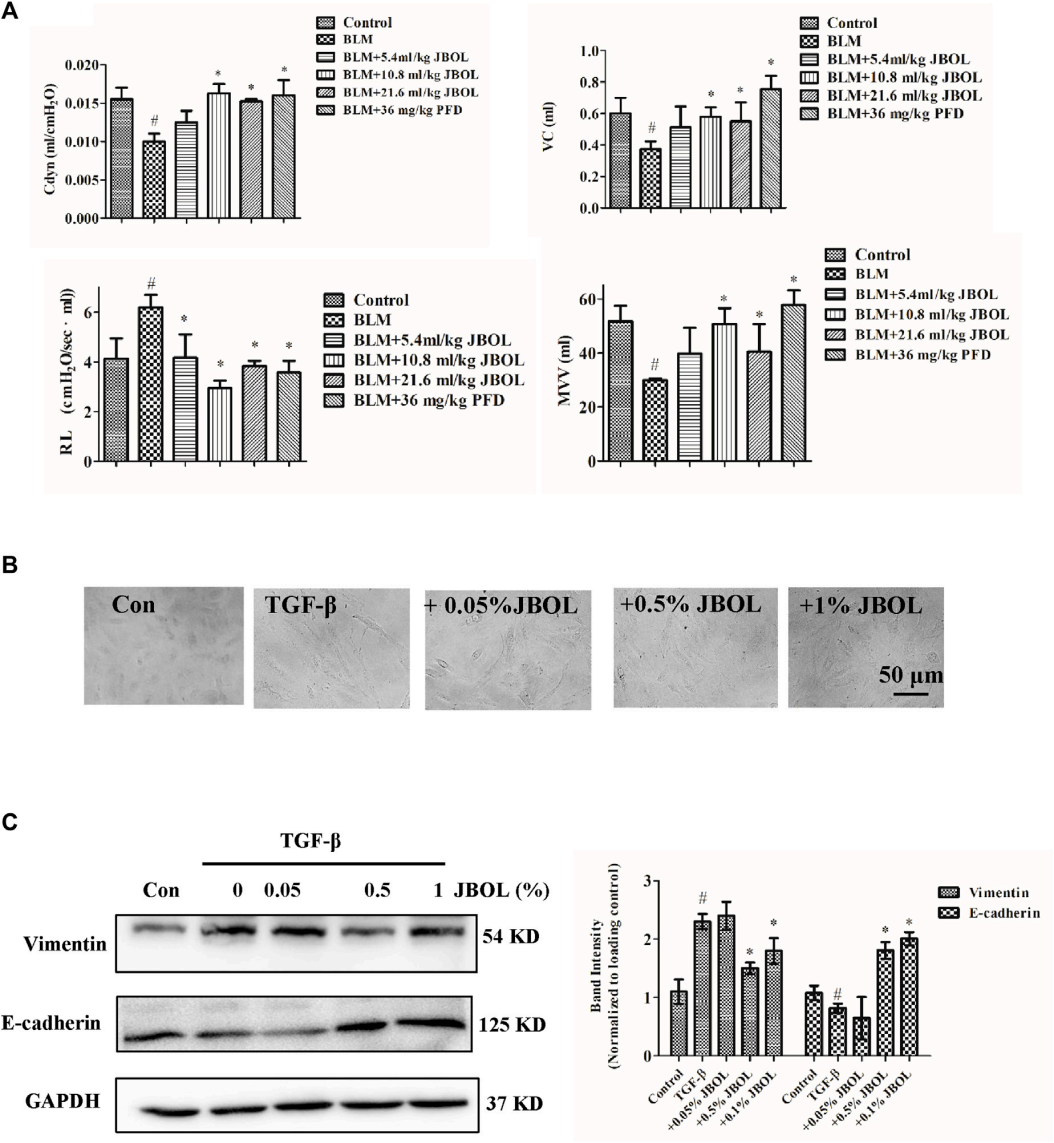
Evaluation of JBOL effects on IPF *in vivo* and *in vitro*. **(A)** Pulmonary function test in the BLM-treated rats with JBOL or pirfenidone (PFD). n = 3–6, *p < 0.05. **(B)** Morphological changes in A549 cells treated with TGF-β1 and/or JBOL. **(C)** The expression of vimentin and E-cadherin. n = 3, #*p* < 0.05 vs control; *p < 0.05 vs the TGF-β1-treated cells.

To test for efficacy, rats were treated with BLM to establish the IPF model, and the effect of JBOL was evaluated. Compared with the BLM group, the Cdyn, MVV, and VC indices increased in the presence of JBOL or PFD, and the RL index decreased with treatment with JBOL or PFD (Figure 8A). These results indicated that JBOL reduced pulmonary functional damage in BLM-induced rats.

TGF-β1 is a critical cytokine capable of inducing epithelial-to-mesenchymal transitions (EMT) during pulmonary fibrosis (Zhang, Fan et al., 2021). We evaluated whether JBOL inhibited TGF-β1–induced EMT in A549 cells. The JBOL dose, which was less than a 1% solution, did not exhibit cytotoxicity. As seen in Figure 8B, when the A549 cells were treated with TGF-β1, the cell shape-shifted from an epithelial form to a spindle-like fibroblast shape, representing the typical morphological characteristics of EMT. The addition of JBOL inhibited the TGF-β1–induced change in cellular morphology. Also, the expression of the mesenchymal marker vimentin was suppressed, and the expression of the epithelial marker E-cadherin increased with JBOL treatment (Figure 8C), indicating that JBOL inhibited the EMT process.

### JBOL Inhibited MAPK ERK1/2, p38, and JNK Phosphorylation in BLM-Induced Rats

**FIGURE 9:**
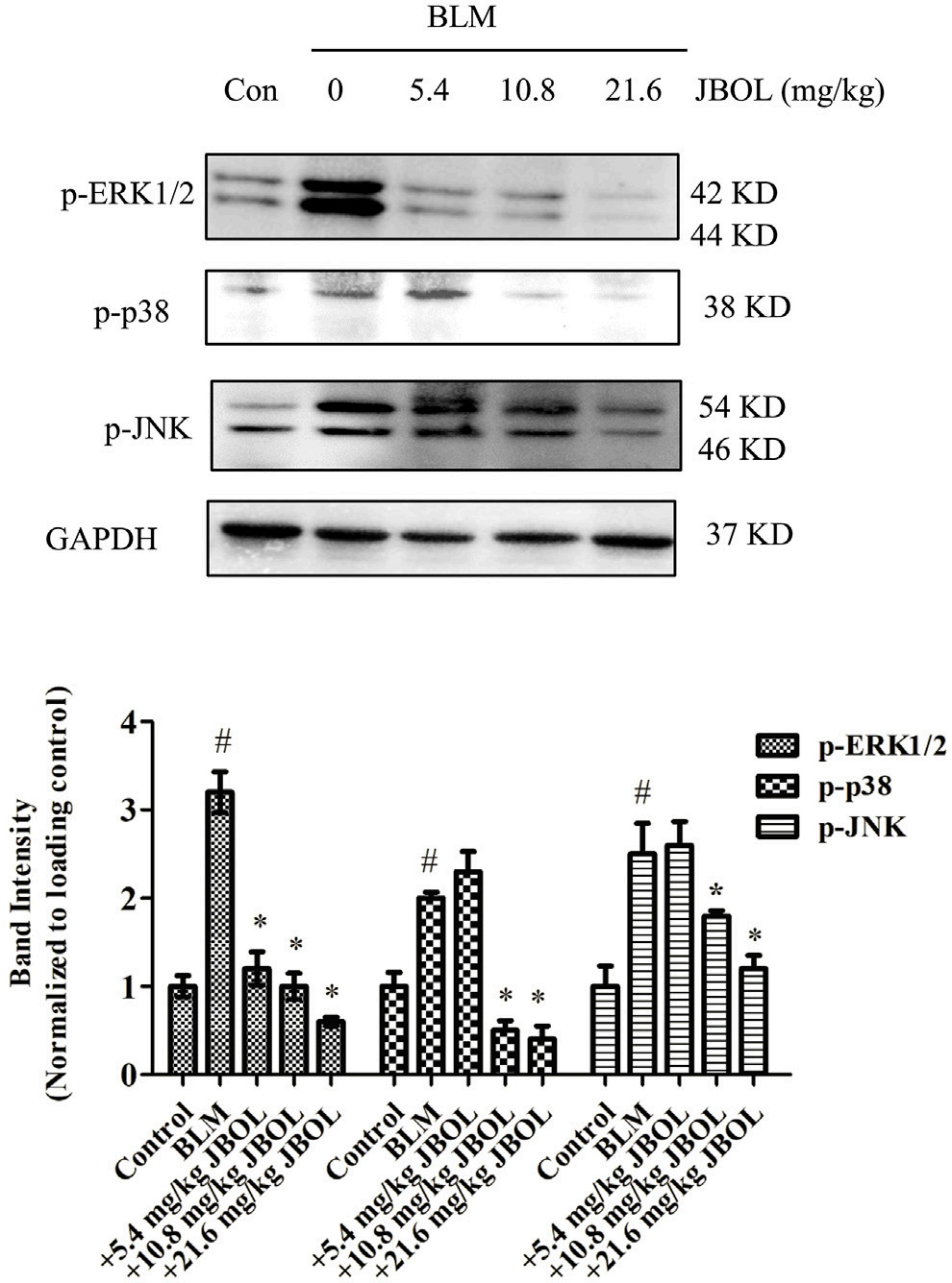
Effect of MAPK phosphorylation *in vivo*. The expression of p-ERK1/2, p-P38, and p-JNK was determined using the Western blot analysis. n = 3–6, #*p* < 0.05 vs. control; *p < 0.05 vs. the BLM-treated group.

Since the hub nodes in the PPI network included MAPK proteins, and MAPK pathways are thought to act downstream of many of the aforementioned identified pathways (Antoniou, Margaritopoulos et al., 2010; Wang, Huang et al., 2018), the phosphorylation levels of extracellular signal-regulated kinase 1/2 (ERK1/2), p38, and c-Jun N-terminal protein kinase (JNK) were evaluated in JBOL-treated rats. The addition of JBOL reduced the increase in phosphorylation levels of ERK1/2, p38, and JNK induced by BLM in a dose-dependent manner (Figure 9). These results were consistent with the network pharmacological analysis suggesting that the MAPK proteins might be central to the JBOL mechanism of action that prevented IPF.

## Discussion

IPF is a chronic, progressive pulmonary disease with an unclear etiology and the pathological characteristics of progressive EMT transition, excessive extracellular matrix protein deposition, and, ultimately, respiratory system failure and death (Wilson and Wynn 2009; Degryse and Lawson 2011). Although several anti-inflammatory and immunomodulatory drugs are available to treat IPF, they fail to prevent its progression and exhibit adverse side effects (Walter and N. 2006; Michihito, Shinjiro et al., 2018). Therefore, novel drugs need to be developed.

JBOL currently is prescribed based on traditional Chinese medicine theory and has been used as a clinical treatment for interstitial lung disease (Tao K 1997). Recently, we reported that JBOL inhibited BLM-induced IPF by regulating the release of inflammatory factors, such as IFN-γ and IL-4 (Zhang, Cui et al., 2018). In this study, the protective effect of JBOL from pulmonary function impairment was observed. Furthermore, the underlying therapeutic mechanism of JBOL for IPF treatment was elucidated. However, the complicated characteristics of traditional Chinese medicine make it challenging to elucidate the underlying actions and mechanisms using the current reductionism research strategy of one gene, one target, and one compound.

In this study, systematic network pharmacology was used to discover the pharmacological mechanisms of JBOL underlying the IPF treatment. The process included JBOL chemical component screening, compound–target prediction, network construction, and experimental validation. Two hundred seventy-eight identified compounds in JBOL targeted 374 proteins. Among these targets, 103 targets were strongly associated with IPF (King, Pardo et al., 2011; Qiu, Wang et al., 2020). The key node targets that included many more edges, possibly have critical roles in the JBOL treatment of IPF, including MAPK1 (ERK2), MAPK14 (p38), MAPK8 (JNK1), JUN, IL-6, IL-4, and AKT. These targets corresponded to steroid hormones, oxygen levels, lipopolysaccharide, and responses to growth factors (Glass, Grossfeld et al., 2020). Il-4 has been validated as a target for JBOL, as we previously reported. Also, IFN-γ was inhibited by JBOL (Zhang, Cui et al., 2018). Additionally, JBOL has been reported to regulate the ratio of Th1/Th2 during chronic lung fibrosis (Xing, Qiang et al., 2020). In this study, additional targets for JBOL in response to the IPF-related inflammation and immune response were revealed.

There are numerous chemical components in JBOL. The active components are challenging to identify and were confirmed by the network pharmacology analysis. Therefore, we further identified major chemical components and determined their content in JBOL using UHPLC. By combining information from the TCMSP database and the UHPLC analysis, seven identified components, guanosine, formononetin, liquiritigenin, isoliquiritigenin, imperatorin, tanshinone I, and 18β-glycyrrhetinic acid, were confirmed as major active components. Although the content of most of the identified compounds was low, the efficacy of JBOL might be attributed to the combined and additive effects of these compounds.

Chlorogenic acid is thought to prevent IPF but has poor oral bioavailability (Wang, Dong et al., 2017). Isoliquiritigenin suppresses fibrogenesis through the PI3K/AKT/mTOR pathway in TGF-β1–treated human lung fibroblast-derived MRC-5 cells (He, Peng et al., 2020). Imperatorin exhibited anti-inflammatory properties on alveolar macrophages associated with pulmonary injury (Li, Chen et al., 2019). In addition, formononetin and tanshinone I target several signaling molecules related to IPF (Ma, Ji et al., 2013; Tao, Zheng et al., 2013); therefore, these components might have essential roles in treating IPF with JBOL. Although 278 compounds were identified using the TCMSP database, not all of these compounds were present in JBOL. We identified 15 compounds in JBOL using the UHPLC analysis. Additional compounds need to be identified in JBOL using the chemical analysis, such as UPLC/MS. Therefore, additional investigation of the active components in JBOL would be beneficial.

We performed an *in vivo* experiment to confirm that JBOL protected pulmonary function in BLM-induced rats. Previously, a histopathological assessment was performed to verify the protective effect of JBOL in the BLM-induced lung impairment (Zhang, Cui et al., 2018; Xing, Qiang et al., 2020). In this study, the protective role of JBOL in pulmonary function was confirmed. We also used TGF-β1 to treat A549 cells to create an *in vitro* IPF model (Oh, Kim et al., 2020). The EMT is critical in the pathology of IPF (Zhang, Fan et al., 2021), and JBOL exposure resulted in a decrease in the spindle-like fibroblast cell shape induced by TGF-β1. EMT markers, including decreased expression of E-cadherin and increased expression of vimentin induced by TGF-β1, were reversed by the JBOL treatment, suggesting that JBOL could inhibit the EMT process in alveolar epithelial cells. The inhibitory effect on EMT in the cultured cells was consistent with a previous study demonstrating JBOL inhibition of EMT in the BLM-treated rats (Zhang, Cui et al., 2018). Also, the compounds in JBOL exhibited immune-modulatory, anti-inflammatory, and antifibrosis activities (Lin and Yang 2021; Yao, Ren et al., 2021). Combined with our previous study, the current results suggested that JBOL prevented IFP by reducing inflammation, fibrosis, and EMT.

To elucidate the molecular mechanism of action of JBOL in the treatment of IPF further, the GO and KEGG pathway analyses were performed to screen for critical candidate targets. The GO analysis revealed several biological processes, including responses to steroid hormones, growth factors, and lipopolysaccharide. The results of the KEGG pathway analysis indicated that a possible mechanism of JBOL for treating IPF involved the regulation of multiple signaling pathways related to inflammation, immunoregulation, and EMT, including MAPK, PI3K/Akt, and HIF-1 signaling pathways as well as others. Aberrant activation of the PI3K/Akt, HIF, and estrogen pathways contribute to fibroblast proliferation, alveolar epithelial cell apoptosis, and EMT (Lu, Azad et al., 2010; Elliot, Periera-Simon et al., 2019). The NF-κB pathway has been suggested as a therapeutic target in IPF (Jaffar J et al., 2021). NF-κB is capable of regulating IFN-γ action in lung fibroblasts through the Fas pathway (Wynes, MW et al., 2011). We proposed that JBOL inhibited IFN-γ possibly through targeting the NF-κB pathway. The cGMP–PKG pathway was involved in autophagy in BLM-induced IPF (Wang Y 2021). Thyroid hormone could inhibit lung fibrosis by improving epithelial mitochondrial function (Yu, Tzouvelekis et al., 2018), and BLM-mediated IPF was found to be dependent on the IL-17 pathway (Wilson and Wynn 2009). Increased AGE accelerated aging in fibrotic lung tissue (Machahua, Montes-Worboys et al., 2016).

The involved signaling pathways and molecular targets were varied and difficult to validate using single experiments. Among these pathways, we assumed that the MAPK proteins might play a central role because the signaling proteins of MAPK1 (ERK2), MAPK14 (p38), MAPK8 (JNK1), and JUN were key hub nodes in the PPI network. Simultaneously, they commonly act as downstream molecules of various signaling pathways to execute signaling functions, such as the PI3K/Akt and HIF-1 signaling pathways (Derynck and Zhang 2003; Roux and Blenis 2004). ERK1/2, p38, and JNK are three members of the MAPK protein family (van der Velden, Ye et al., 2016; Goda, Balli et al., 2020) and participate in the pathological mechanism of pulmonary fibrosis (Yu, Song et al., 2019). JNK was activated during the BLM-induced alveolar epithelial cell death (Lee, Schroedl et al., 2005). p38 and ERK were phosphorylated in the TGF-β–induced rat fibroblast NRK-49F cells (Chen, Zhou et al., 2013). MAPK also participated in regulating the production of inflammatory factors, TGF-β/Smads signaling or acted downstream of the PI3K and HIF pathways (Derynck and Zhang 2003), indicating a central role for the MAPK pathway in IPF pathology. In this study, JBOL inhibited the expression of phosphorylated ERK1/2, p38, and JNK in the BLM-treated rats, suggesting that JBOL inhibited the MAPK activation. Thus, we concluded that this might be a central event in the systematic network triggered by JBOL. In the future, the active components of JBOL targeting MAPK proteins and also other targets screened from network pharmacology regulated by JBOL require additional investigation.

## Conclusion

This study is the first to show that JBOL could improve pulmonary function and inhibit EMT in the TGF-β1–induced A549 cells. The pharmacological mechanisms underlying the efficacy of JBOL might be related to systematic network regulation, including multiple components targeting several pathways centered on inhibiting phosphorylation of MAPK ERK1/2, p38, and JNK. Thus, these findings provide a theoretical and experimental basis for promoting the clinical application of JBOL.

## Data Availability

The raw data supporting the conclusions of this article will be made available by the authors, without undue reservation.
