# Supplementary material for: Investigation of the Pharmacological Effect and Mechanism of Jinbei Oral Liquid in the Treatment of Idiopathic Pulmonary Fibrosis Using Network Pharmacology and Experimental Validation
Source: Front Pharmacol. 2022 Jun 15;13:919388. doi: 10.3389/fphar.2022.919388 (PMC9240387; doi:10.3389/fphar.2022.919388)
Supplement: Supplementary file 3 [file Table5.DOCX]

Table 1: Basic information for JBOL compound ingredients

| CAS | Molecule Name | Structure | OB(%)/GI absorption | Druglikeness |
| --- | --- | --- | --- | --- |
| 21754-25-6 | Cyclo-(Val-Tyr) | 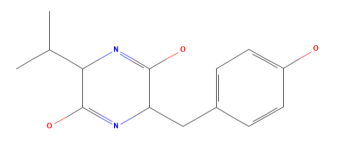 | 122.79 | 0.14 |
| N/A | Forsythidmethylester_qt | 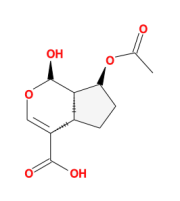 | 121.84 | 0.12 |
| N/A | Cyclo-(Leu-Tyr) | 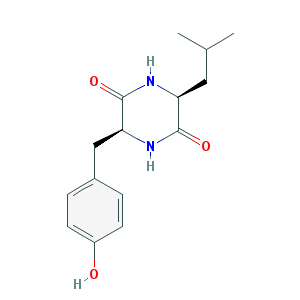 | 111.16 | 0.15 |
| N/A | Shanzhiside  methyl ester_qt | 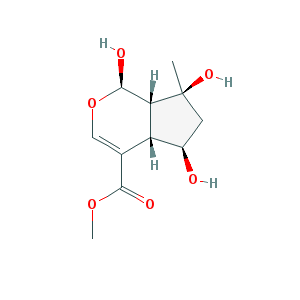 | 109.77 | 0.12 |
| 55084-08-7 | NEOBAICALEIN | 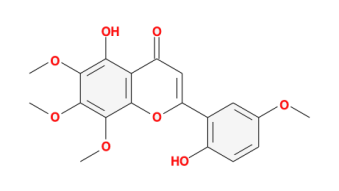 | 104.34 | 0.44 |
| 487-39-8 | (-)-Phillygenin | 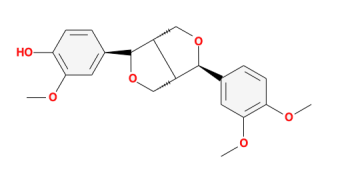 | 95.04 | 0.57 |
| 529-61-3 | Euxanthone | 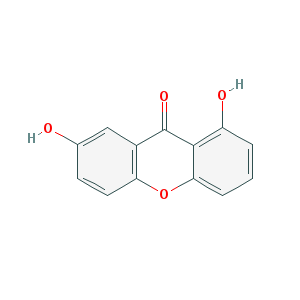 | 92.98 | 0.16 |
| 23013-84-5 | Glycyrol | 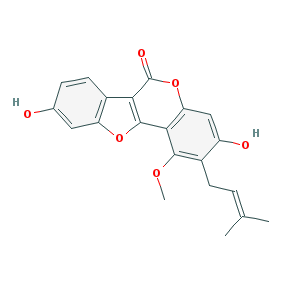 | 90.78 | 0.67 |
| N/A | Loniceracetalide A_qt | 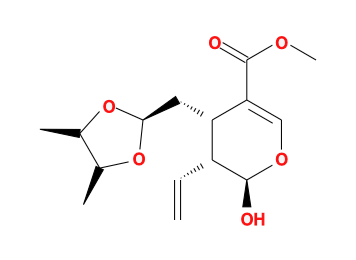 | 89.38 | 0.17 |
| 961-29-5 7014-39-3 | 1. -1-(2,4-dihydroxyphenyl)   -3-(4-hydroxyphenyl)prop-  2-en-1-one | 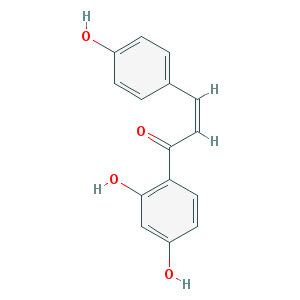 | 87.51 | 0.15 |
| N/A | (-)-(3R,8S,9R,9aS,10aS)-9-ethenyl-8-(beta-D-glucopyranosyloxy)-2,3,9,9a,10,10a-hexahydro-5-oxo-5H,8H-pyrano[4,3-d]oxazolo[3,2-a]pyridine-3-carboxylic acid_qt | 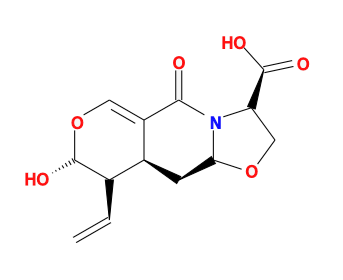 | 87.47 | 0.23 |
| 489-41-8 | 49070_FLUKA | 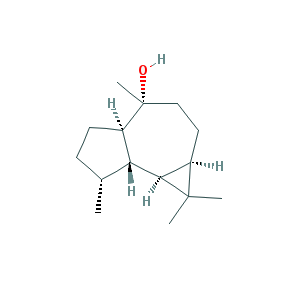 | 85.51 | 0.12 |
| 961-29-5 13745-20-5 | Isoliquiritigenin | 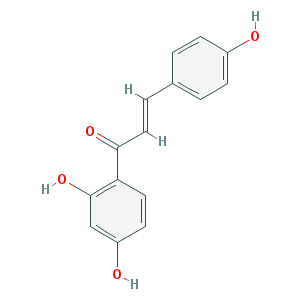 | 85.32 | 0.15 |
| 487-41-2 | ACon1_001697 | 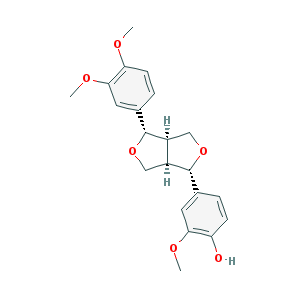 | 85.12 | 0.57 |
| N/A | 7,2',4'-trihydroxy-5-methoxy-3－arylcoumarin | 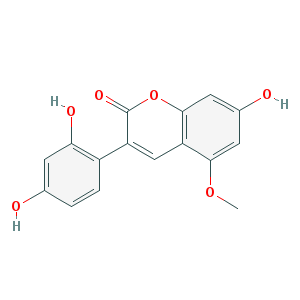 | 83.71 | 0.27 |
| N/A | FORSYTHINOL | 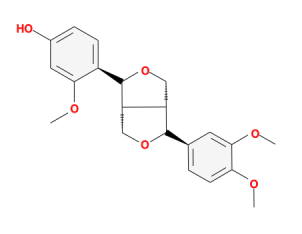 | 81.25 | 0.57 |
| 117038-80-9 | licopyranocoumarin | 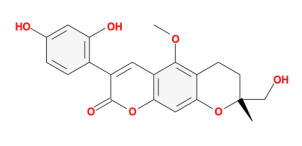 | 80.36 | 0.65 |
| 157414-04-5 | shinpterocarpin | 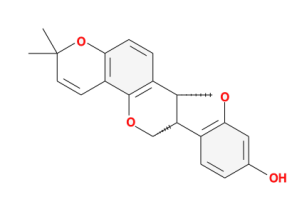 | 80.3 | 0.73 |
| N/A | Onjixanthone I | 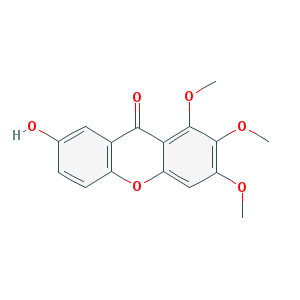 | 79.16 | 0.3 |
| N/A | Phaseol | 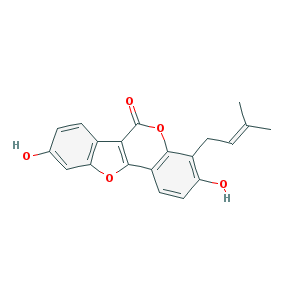 | 78.77 | 0.58 |
| 485-72-3 | formononetin | 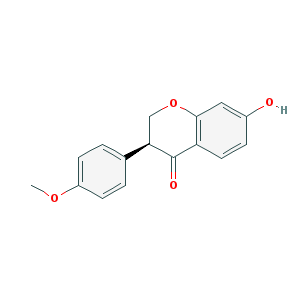 | 69.67 | 0.21 |
| 482-44-0  70102-00-0 | Imperatorin | 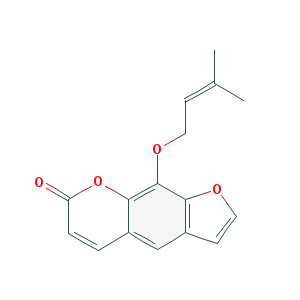 | 34.55 | 0.22 |
| 578-86-9  16006-91-0  17002-53-8  2885-30-5 | Liquiritigenin | 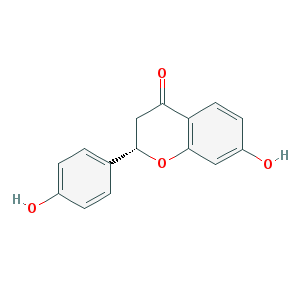 | 32.76 | 0.18 |
| 568-73-0 | Tanshinone I | 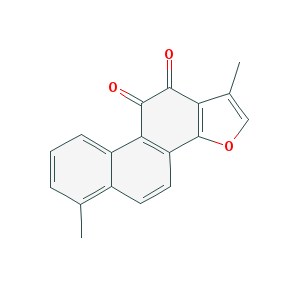 | 29.27 | 0.36 |
| 471-53-4  15301-63-0  8055-71-8  202522-39-2  107420-91-7 | 18β-Glycyrrhetinic Acid | 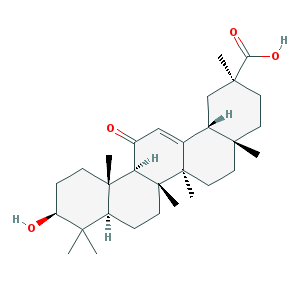 | 22.05 | 0.74 |
| 118-00-3 | guanosine | 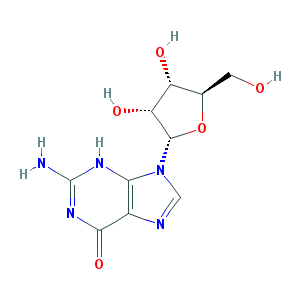 | 21.43 | 0.21 |
