# Supplementary material for: Investigation of the Pharmacological Effect and Mechanism of Jinbei Oral Liquid in the Treatment of Idiopathic Pulmonary Fibrosis Using Network Pharmacology and Experimental Validation
Source: Front Pharmacol. 2022 Jun 15;13:919388. doi: 10.3389/fphar.2022.919388 (PMC9240387; doi:10.3389/fphar.2022.919388)
Supplement: Supplementary file 5 [file Table4.DOCX]

Table 2: Details on potential targets and the topological attributes.

| No. | Gene name | Protein name | UniProt ID | Degree |
| --- | --- | --- | --- | --- |
| 1 | PTGS2 | prostaglandin-endoperoxide synthase 2 | P35354 | 175.0 |
| 2 | HSP90AA1 | heat shock protein 90 alpha family class A member 1 | P07900 | 129.0 |
| 3 | ESR1 | estrogen receptor 1 | P03372 | 107.0 |
| 4 | PTGS1 | prostaglandin-endoperoxide synthase 1 | P23219 | 106.0 |
| 5 | NOS2 | nitric oxide synthase 2 | P35228 | 99.0 |
| 6 | F10 | coagulation factor X | P00742 | 79.0 |
| 7 | PIM1 | Pim-1 proto-oncogene, serine/threonine kinase | P11309 | 74.0 |
| 8 | MAPK14 | mitogen-activated protein kinase 14 | Q16539 | 71.0 |
| 9 | CCNA2 | cyclin A2 | P20248 | 64.0 |
| 10 | PIK3CG | phosphatidylinositol-4,5-bisphosphate 3-kinase catalytic subunit gamma | P48736 | 44.0 |
| 11 | BCL2 | BCL2 apoptosis regulator | P10415 | 21.0 |
| 12 | AKT1 | AKT serine/threonine kinase 1 | P31749 | 18.0 |
| 13 | CASP3 | caspase 3 | P42574 | 18.0 |
| 14 | RELA | RELA proto-oncogene, NF-kB subunit | Q04206 | 18.0 |
| 15 | KCNMA1 | potassium calcium-activated channel subfamily M alpha 1 | Q12791 | 17.0 |
| 16 | JUN | Jun proto-oncogene, AP-1 transcription factor subunit | P05412 | 15.0 |
| 17 | MAPK1 | mitogen-activated protein kinase 1 | P28482 | 14.0 |
| 18 | TP53 | tumor protein p53 | P04637 | 13.0 |
| 19 | HTR2A | 5-hydroxytryptamine receptor 2A | P28223 | 13.0 |
| 20 | FOS | Fos proto-oncogene, AP-1 transcription factor subunit | P01100 | 12.0 |
| 21 | CDKN1A | cyclin dependent kinase inhibitor 1A | P38936 | 10.0 |
| 22 | CCND1 | cyclin D1 | P24385 | 10.0 |
| 23 | RAF1 | Raf-1 proto-oncogene, serine/threonine kinase | P04049 | 10.0 |
| 24 | ICAM1 | intercellular adhesion molecule 1 | P05362 | 10.0 |
| 25 | NR3C1 | nuclear receptor subfamily 3 group C member 1 | P04150 | 10.0 |
| 26 | IL6 | interleukin 6 | P05231 | 9.0 |
| 27 | VEGFA | vascular endothelial growth factor A | P15692 | 9.0 |
| 28 | HMOX1 | heme oxygenase 1 | P09601 | 9.0 |
| 29 | LTA4H | leukotriene A4 hydrolase | P09960 | 9.0 |
| 30 | TGFB1 | transforming growth factor beta 1 | P01137 | 8.0 |
| 31 | IFNG | interferon gamma | P01579 | 8.0 |
| 32 | SERPINE1 | serpin family E member 1 | P05121 | 8.0 |
| 33 | MMP9 | matrix metallopeptidase 9 | P14780 | 8.0 |
| 34 | EGFR | epidermal growth factor receptor | P00533 | 8.0 |
| 35 | MMP1 | matrix metallopeptidase 1 | P03956 | 8.0 |
| 36 | EDN1 | endothelin 1 | P05305 | 8.0 |
| 37 | HIF1A | hypoxia inducible factor 1 subunit alpha | Q16665 | 7.0 |
| 38 | CXCL8 | C-X-C motif chemokine ligand 8 | P10145 | 7.0 |
| 39 | IL4 | interleukin 4 | P05112 | 7.0 |
| 40 | VCAM1 | vascular cell adhesion molecule 1 | P19320 | 7.0 |
| 41 | FASLG | Fas ligand | P48023 | 7.0 |
| 42 | STAT3 | signal transducer and activator of transcription 3 | P40763 | 6.0 |
| 43 | PLAU | plasminogen activator, urokinase | P00749 | 6.0 |
| 44 | TNF | tumor necrosis factor | P01375 | 6.0 |
| 45 | ACTB | actin beta | P60709 | 6.0 |
| 46 | IL1B | interleukin 1 beta | P01584 | 6.0 |
| 47 | CTNNB1 | catenin beta 1 | P35222 | 6.0 |
| 48 | EGF | epidermal growth factor | P01133 | 6.0 |
| 49 | MAPK8 | mitogen-activated protein kinase 8 | P45983 | 6.0 |
| 50 | AHSA1 | activator of HSP90 ATPase activity 1 | O95433 | 6.0 |
| 51 | MMP2 | matrix metallopeptidase 2 | P08253 | 6.0 |
| 52 | IL2 | interleukin 2 | P60568 | 6.0 |
| 53 | CCL2 | C-C motif chemokine ligand 2 | P13500 | 5.0 |
| 54 | IL1A | interleukin 1 alpha | P01583 | 5.0 |
| 55 | PTEN | phosphatase and tensin homolog | P60484 | 5.0 |
| 56 | NR1I2 | nuclear receptor subfamily 1 group I member 2 | O75469 | 5.0 |
| 57 | SPP1 | secreted phosphoprotein 1 | P10451 | 5.0 |
| 58 | JAK2 | Janus kinase 2 | O60674 | 5.0 |
| 59 | COL1A1 | collagen type I alpha 1 chain | P02452 | 5.0 |
| 60 | ELK1 | ETS transcription factor ELK1 | P19419 | 5.0 |
| 61 | ERBB2 | erb-b2 receptor tyrosine kinase 2 | P04626 | 5.0 |
| 62 | IGF1R | insulin like growth factor 1 receptor | P08069 | 5.0 |
| 63 | FOSL2 | FOS like 2, AP-1 transcription factor subunit | P15408 | 4.0 |
| 64 | CAV1 | caveolin 1 | Q03135 | 4.0 |
| 65 | FN1 | fibronectin 1 | P02751 | 4.0 |
| 66 | RASA1 | RAS p21 protein activator 1 | P20936 | 4.0 |
| 67 | IL13 | interleukin 13 | P35225 | 4.0 |
| 68 | IL10 | interleukin 10 | P22301 | 4.0 |
| 69 | MPO | myeloperoxidase | P05164 | 4.0 |
| 70 | XIAP | X-linked inhibitor of apoptosis | P98170 | 4.0 |
| 71 | COL3A1 | collagen type III alpha 1 chain | P02461 | 4.0 |
| 72 | BDNF | brain derived neurotrophic factor | P23560 | 4.0 |
| 73 | BMPR2 | bone morphogenetic protein receptor type 2 | Q13873 | 4.0 |
| 74 | ALOX5 | arachidonate 5-lipoxygenase | P09917 | 4.0 |
| 75 | MMP3 | matrix metallopeptidase 3 | P08254 | 3.0 |
| 76 | NFE2L2 | nuclear factor, erythroid 2 like 2 | Q16236 | 3.0 |
| 77 | PLG | plasminogen | P00747 | 3.0 |
| 78 | CDKN2A | cyclin dependent kinase inhibitor 2A | P42771;Q8N726 | 3.0 |
| 79 | CXCL10 | C-X-C motif chemokine ligand 10 | P02778 | 3.0 |
| 80 | CRP | C-reactive protein | P02741 | 3.0 |
| 81 | PCNA | proliferating cell nuclear antigen | P12004 | 3.0 |
| 82 | DDIT3 | DNA damage inducible transcript 3 | P35638 | 3.0 |
| 83 | CFLAR | CASP8 and FADD like apoptosis regulator | O15519 | 3.0 |
| 84 | ABCG2 | ATP binding cassette subfamily G member 2 (Junior blood group) | Q9UNQ0 | 3.0 |
| 85 | CXCL11 | C-X-C motif chemokine ligand 11 | O14625 | 3.0 |
| 86 | SOAT1 | sterol O-acyltransferase 1 | P35610 | 3.0 |
| 87 | SIRT1 | sirtuin 1 | Q96EB6 | 3.0 |
| 88 | PARP1 | poly(ADP-ribose) polymerase 1 | P09874 | 3.0 |
| 89 | MMP13 | matrix metallopeptidase 13 | P45452 | 3.0 |
| 90 | HSPB1 | heat shock protein family B (small) member 1 | P04792 | 3.0 |
| 91 | BIRC5 | baculoviral IAP repeat containing 5 | O15392 | 3.0 |
| 92 | HK2 | hexokinase 2 | P52789 | 3.0 |
| 93 | TERT | telomerase reverse transcriptase | O14746 | 2.0 |
| 94 | MUC1 | mucin 1, cell surface associated | P15941 | 2.0 |
| 95 | TIMP3 | TIMP metallopeptidase inhibitor 3 | P35625 | 2.0 |
| 96 | HSPA5 | heat shock protein family A (Hsp70) member 5 | P11021 | 2.0 |
| 97 | IGFBP3 | insulin like growth factor binding protein 3 | P17936 | 2.0 |
| 98 | E2F1 | E2F transcription factor 1 | Q01094 | 2.0 |
| 99 | ADIPOQ | adiponectin, C1Q and collagen domain containing | Q15848 | 2.0 |
| 100 | RUNX2 | RUNX family transcription factor 2 | Q13950 | 2.0 |
| 101 | ECE1 | endothelin converting enzyme 1 | P42892 | 2.0 |
| 102 | ALPI | alkaline phosphatase, intestinal | P09923 | 2.0 |
| 103 | CYP2C9 | cytochrome P450 family 2 subfamily C member 9 | P11712 | 2.0 |
